# Intravascular Ultrasound to Assess Transcatheter Heart Valve Geometry During Aortic Valve-In-Valve Procedure

**DOI:** 10.1016/j.shj.2026.101131

**Published:** 2026-09-11

**Authors:** Radosław Pracoń, Maciej Dąbrowski, Yusuke Kobari, Ania Pyłko, Arif Khokhar, Jacek Kądziela, Karol A. Sadowski, Michał Kania, Marcin Demkow, Ole De Backer, Łukasz Kalińczuk

**Affiliations:** a National Institute of Cardiology, Warsaw, Poland; b Københavns Universitet, The Heart Center – Rigshospitalet, Copenhagen, Denmark

**Keywords:** Intravascular ultrasound, Transcatheter aortic valve replacement, Transcatheter heart valve expansion, Valve-in-valve

A 68-year-old man with a degenerated 23-mm Perimount surgical aortic bioprosthesis (Edwards Lifesciences, Irvine, California) was accepted for transfemoral valve-in-valve transcatheter aortic valve replacement. Although a 23-mm Navitor transcatheter heart valve (THV) (Abbott, Santa Clara, California) would provide 10% oversizing relative to the 21-mm true inner diameter, planned Perimount fracture with a 24-mm Atlas Gold balloon (Becton Dickinson, Tempe, Arizona) prompted selection of a 25-mm Navitor. Because THV underexpansion after valve-in-valve transcatheter aortic valve replacement is common and may impair hemodynamics, intraprocedural assessment of the final result may be clinically relevant.^1^

Navitor stent-frame dimensions were measured by angiography and intravascular ultrasound (IVUS) at inflow, leaflet nadir, and leaflet coaptation; Perimount ring dimensions were also measured. IVUS was performed using a 15-MHz OptiCross 35 catheter (Boston Scientific, Marlborough, Massachusetts) over the standard 0.035-inch stiff guidewire and measured inner-frame minimum diameter (D_min_), maximum diameter, cross-sectional area (CSA), and geometric orifice area (GOA). Attention was paid to alignment parallel to the THV long axis because oblique acquisition may preferentially affect maximum diameter and CSA, whereas D_min_ is less affected.^2^

After Navitor implantation, the available angiographic projection showed subnominal frame dimensions. Compared with IVUS D_min_, angiographic diameters were larger by 2.62–3.60 mm, corresponding to 10.5–14.4 percentage-point differences in calculated expansion (Figure 1). After Atlas postdilatation, these differences narrowed to 0.61–1.07 mm and 2.4–4.3 percentage points, respectively. At inflow, the circular-equivalent area inferred from angiography differed from directly measured IVUS CSA by only 0.4 percentage points. Concordance was not uniform across THV levels: at the leaflet nadir, angiography implied 75.3% area expansion vs. 67.6% by IVUS, whereas at coaptation it implied 84.5% vs. 93.3%, corresponding to 7.7-point and 8.8-point discrepancies (Figure 2). Dynamic IVUS further showed that GOA increased from 250.88 mm^2^ to 366.77 mm^2^ (+46.2%) and leaflet folding decreased after postdilatation. Despite 93.3% coaptation-site frame expansion (CSA 458.16 mm^2^), peak-systolic GOA occupied only 80.0% of the surrounding frame lumen and remained only moderately larger than the constrained inflow CSA (∼307 mm^2^) (Figure 3).

**Figure 1:**
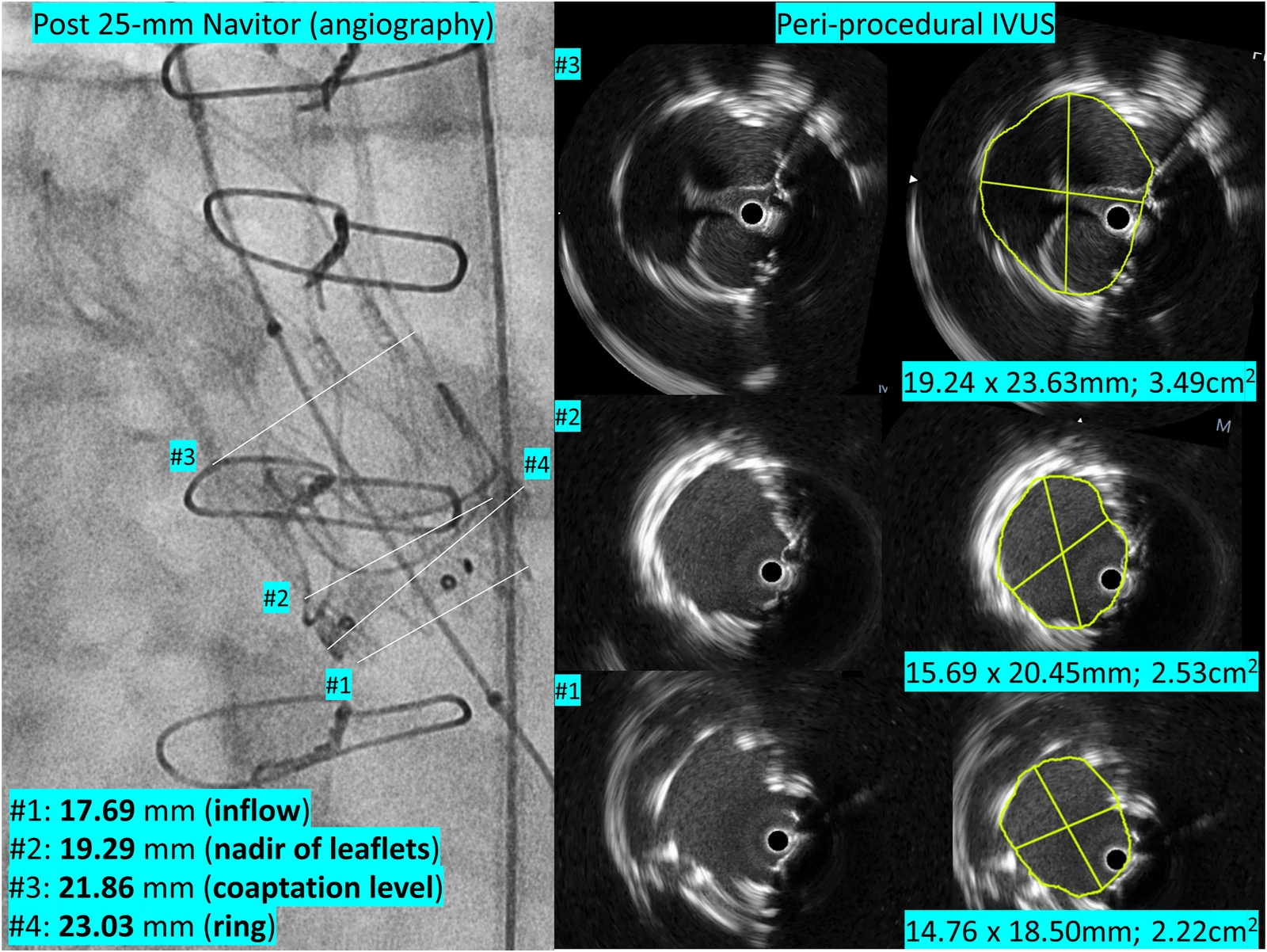
**After 25-mm Navitor deployment, angiographic diameters at inflow, leaflet nadir, and coaptation were 17.69mm, 19.29mm, and 21.86 mm, corresponding to 70.8%, 77.2%, and 87.4% projected expansion, respectively.** Corresponding IVUS D_min_ values were 14.76 mm, 15.69 mm, and 19.24 mm, representing 59.0%, 62.8%, and 77.0% expansion, whereas IVUS CSA expansion was 45.2%, 51.5%, and 71.1%, respectively. Abbreviations: CSA, cross-sectional area; D_min_ minimum diameter; IVUS, intravascular ultrasound.

**Figure 2:**
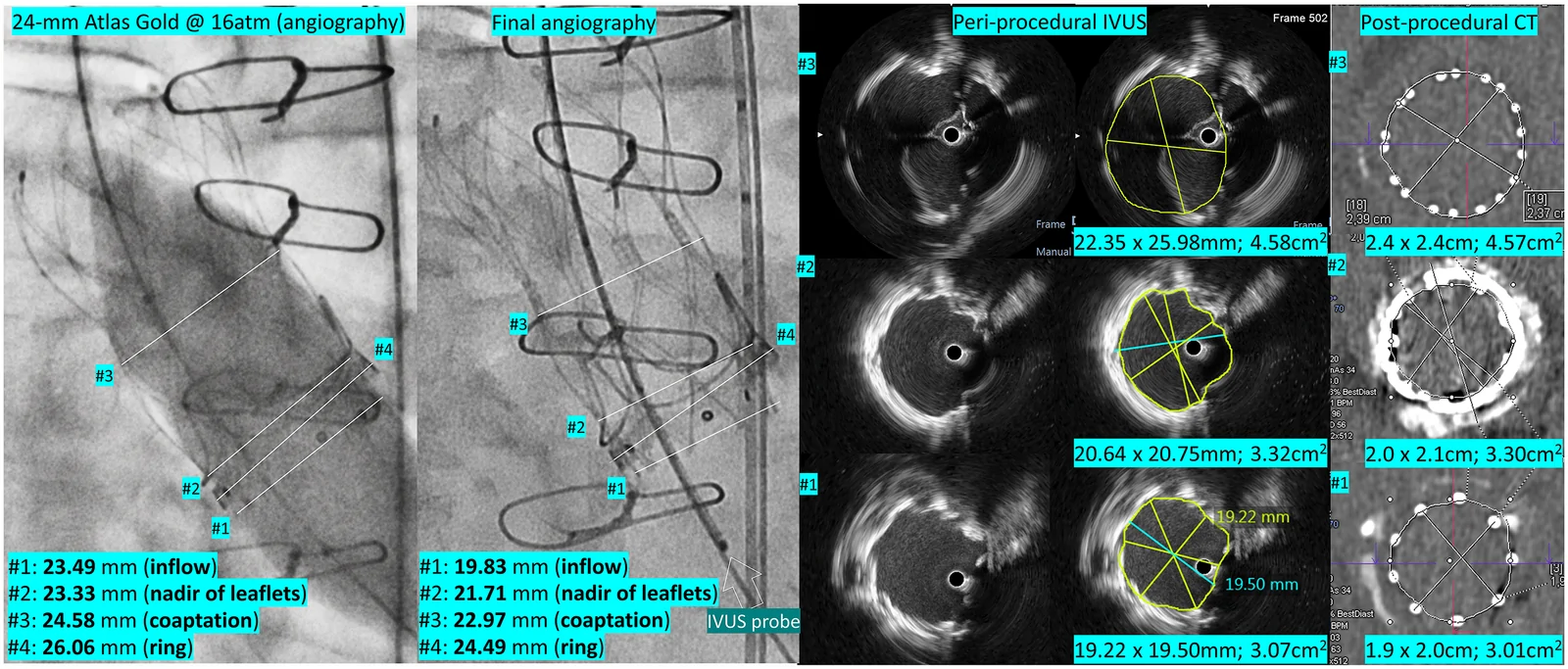
**During 24-mm Atlas Gold balloon inflation, angiography showed Perimount ring expansion to 26.06 mm followed by recoil to 24.49 mm.** Final Navitor diameters at inflow, leaflet nadir, and coaptation were 19.83 mm, 21.71 mm, and 22.97 mm by angiography, corresponding to 79.3%, 86.8%, and 91.9% projected expansion, respectively. Corresponding IVUS D_min_ values were 19.22 mm, 20.64 mm, and 22.35 mm, representing 76.9%, 82.6%, and 89.4% expansion, whereas IVUS CSA expansion was 62.5%, 67.6%, and 93.3%, respectively. Abbreviations: CSA, cross-sectional area; CT, computed tomography; D_min_ minimum diameter; IVUS, intravascular ultrasound.

**Figure 3:**
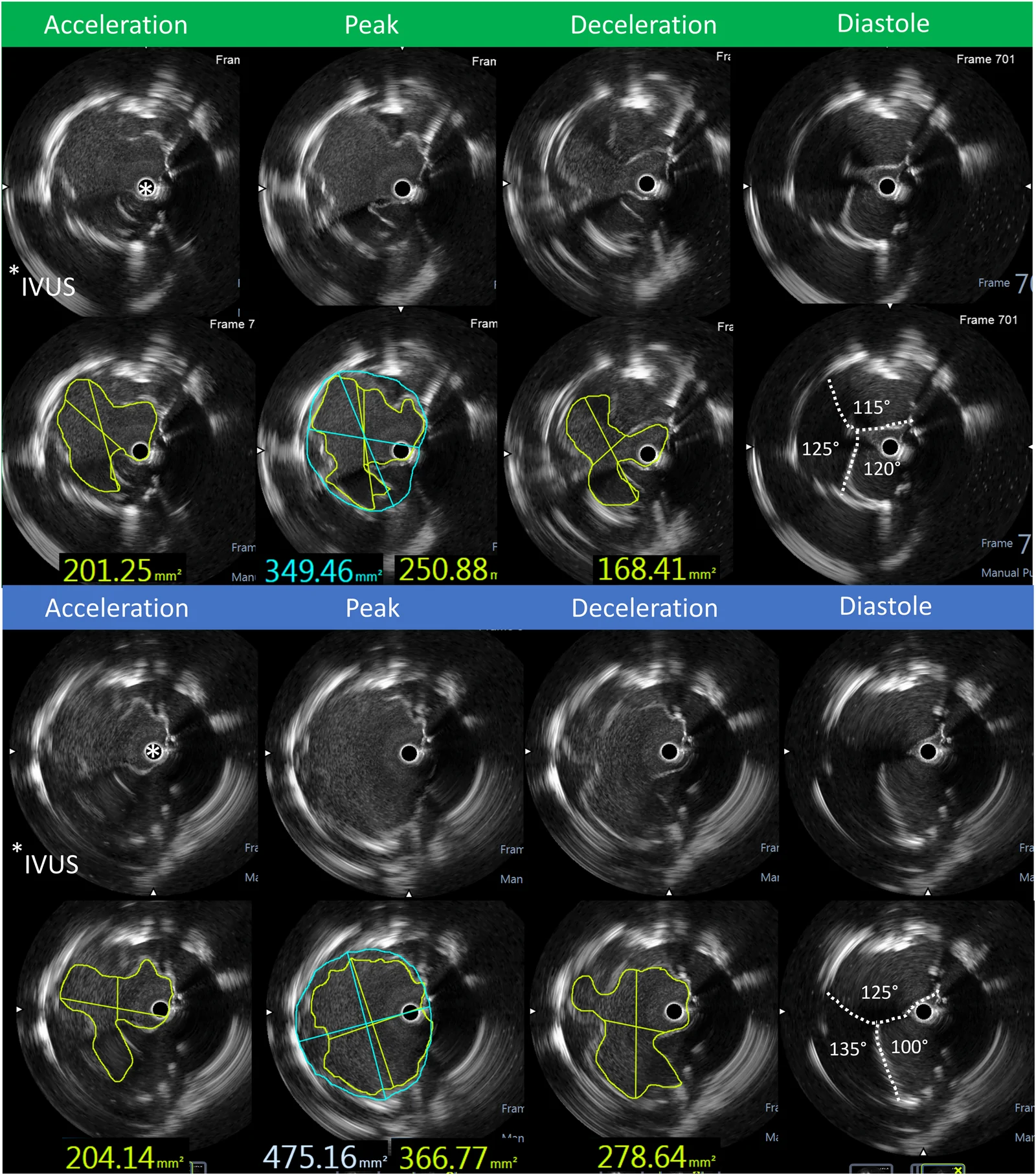
**In a separate dynamic leaflet acquisition, IVUS demonstrated leaflet margins and GOA after Navitor deployment and after Atlas postdilatation.** Postdilatation increased peak-systolic GOA and reduced leaflet folding; measured frame and leaflet-orifice values are displayed in the panels. Abbreviations: GOA, geometric orifice area; IVUS, intravascular ultrasound.

At follow-up, transthoracic echocardiography showed no aortic regurgitation and a mean transprosthetic gradient of 11 mmHg. Postprocedural computed tomography confirmed Perimount ring fracture with slight enlargement and demonstrated close quantitative agreement with IVUS (Figures 3 and 4).

**Figure 4:**
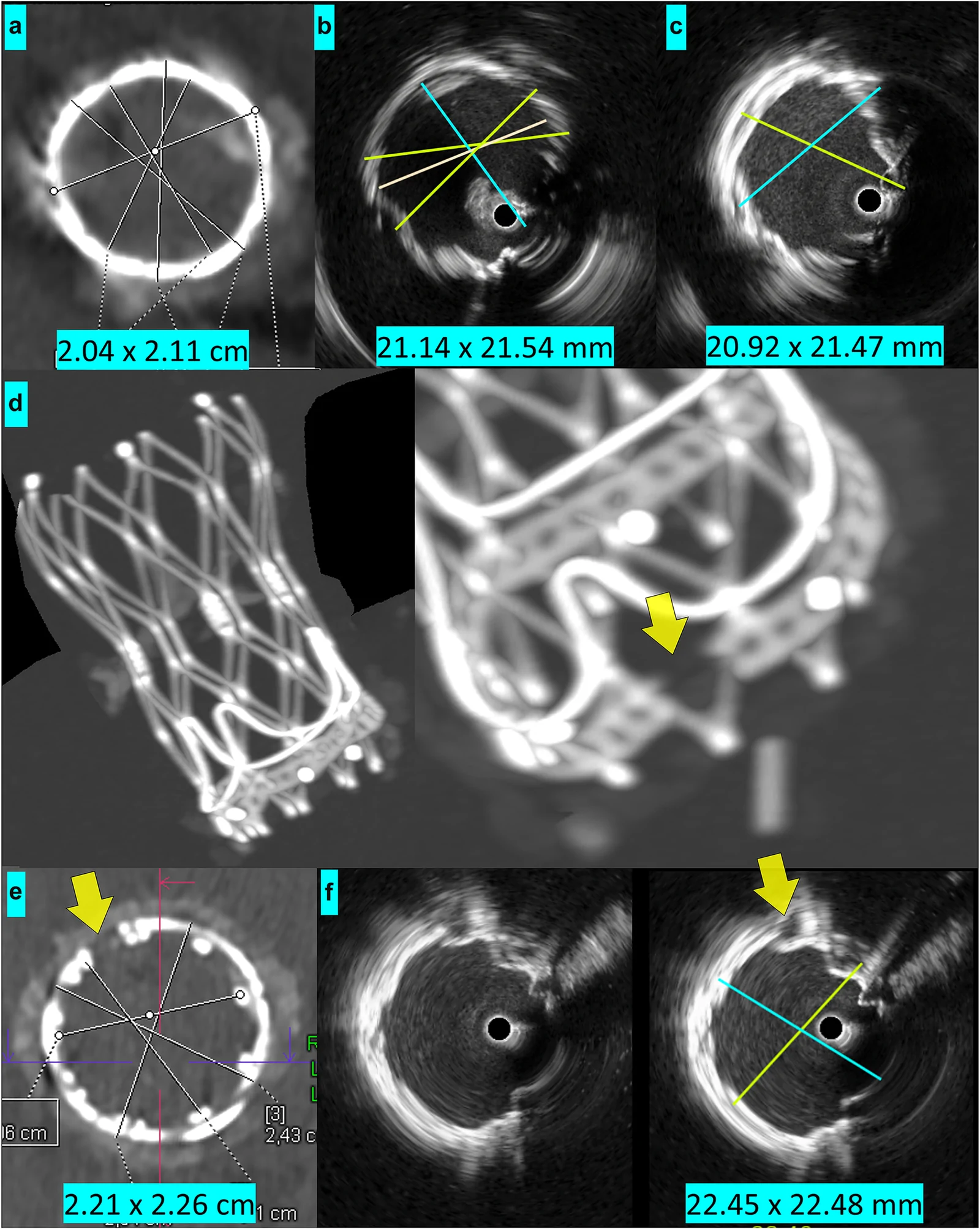
**Baselinecomputed tomographyand periprocedural IVUS showed similar Perimount ring dimensions.** (a) Baseline CT measurement of the Perimount inner ring. (b) Corresponding baseline IVUS measurement. (c) IVUS measurement after valve implantation and before ring fracture. (d) Angiographic appearance after high-pressure balloon inflation, with subtle evidence of ring fracture. (e) Postprocedural CT confirming ring fracture (arrow) and slight enlargement of the inner ring. (f) Final IVUS demonstrating corresponding enlargement of the Perimount ring after fracture and recoil (arrow). Abbreviations: CT, computed tomography; IVUS, intravascular ultrasound.

Angiography remains essential for device position and gross frame expansion; however, even orthogonal projections depict only 2-dimensional frame deformation and cannot directly visualize the leaflet-defined systolic orifice. This limitation may be amplified in valve-in-valve procedures by superimposition of the surgical and transcatheter frames. In this case, postdilatation substantially improved angiography–IVUS diameter concordance, yet tomographic IVUS continued to demonstrate level-dependent, nonuniform geometry. More importantly, dynamic IVUS showed that near-nominal coaptation-level frame expansion did not translate into an equivalently large systolic leaflet opening. Thus, a satisfactory frame silhouette does not establish the size or configuration of the functional orifice. If angiography and hemodynamics alone had been used, stopping after Atlas would have been reasonable; conversely, residual lower-frame constraint could have prompted further optimization. IVUS therefore added mechanistic information in this procedural gray zone, supporting completion without further balloon escalation, although this case cannot establish that management would have differed without IVUS. GOA is a geometric determinant, not a direct measurement, of transvalvular flow; the favorable follow-up gradient provides complementary functional corroboration. Prospective evaluation is required to establish clinical benefit and cost-effectiveness.

## Ethics Statement

The study protocol complied with the Declaration of Helsinki and was approved by the Institutional Review Board of the National Institute of Cardiology, Warsaw, Poland.

## Consent Statement

Written informed consent was obtained from the patient for the publication of this report and accompanying images.

## Funding

This work was supported by research grant 2.15/VI/26.

## Declaration of Generative AI and AI-Assisted Technologies in the Writing Process

ChatGPT and Gemini were minimally used during the manuscript preparation process to assist with improving language clarity, grammar, and structure. Afterward, senior authors reviewed the manuscript and gave final approval. All scientific content, analysis, and final decisions regarding the manuscript were solely conducted and approved by the authors, ensuring the intellectual and academic rigor of the work.

## Disclosure Statement

The authors report no conflict of interest.
